# “Realizing the problem wasn’t necessarily me”: the meaning of childhood adversity and resilience in the lives of autistic adults

**DOI:** 10.1080/17482631.2022.2051237

**Published:** 2022-03-17

**Authors:** Gabrielle A. Heselton, Gwen R. Rempel, David B. Nicholas

**Affiliations:** a Faculty of Health Disciplines (FHD), Athabasca University, Athabasca, Alberta, Canada; b Faculty of Social Work, Central and Northern Alberta Region, University of Calgary, Calgary, Alberta, Canada

**Keywords:** Autism, interpretative phenomenological analysis, participatory methods, childhood adversity, resilience, mental health

## Abstract

**Purpose:**

There is evidence that childhood adversity is correlated with poor health outcomes across the lifespan. Resilience results when internal and external protective factors in childhood mitigate this relationship. However, among children on the autism spectrum, these relationships are understudied, and little is known about the characteristics and role of adversity and resilience in their in their lives. This study interprets these phenomena as experienced by autistic young adults.

**Methods:**

Initially, we conducted community engagement with five members of the autism community who advised on the research question, research design, and analysis. Subsequently, four autistic young adults, three women and one non-binary, aged 19–27, were recruited to participate in semi-structured interviews via phone, video conference, and online chat. Credibility checking interviews followed data analysis.

**Results:**

Through interpretative phenomenological analysis we identified themes related to the negative effects of adversity, including *social disconnection, mental and emotional well-being, sense of self*, and *development into young adulthood*. Resilience developed in *places of refuge* and *identity* and was evident in their *transitions into young adulthood*.

**Conclusion:**

These findings provide direction for decreasing adversity and fostering resilience in children and adolescents on the autism spectrum.

## Introduction

Childhood adversity has been established in the literature as a predictor of poor physical and mental health outcomes in adulthood (Bright et al., 2016; Felitti et al., 1998; Hughes et al., 2017). Additionally, there is evidence that adversity is correlated with physical and mental health outcomes for children and adolescents (Bellis et al., 2018; Bright et al., 2016; Rigles, 2017). Adverse experiences are diverse and may include abuse, neglect, bullying, parental death or illness, exposure to violence, and living in poverty (Berg et al., 2016; Felitti et al., 1998; Hughes et al., 2017; Kerns et al., 2015; Mehtar & Mukaddes, 2011; Moore & Ramirez, 2016; Taylor & Gotham, 2016). However, the potential negative effects associated with childhood adversity can be ameliorated by internal and external protective factors—a concept known as *resilience*. (Gartland et al., 2019; Herrman et al., 2011; Liu et al., 2020; Moore & Ramirez, 2016). In relation to experiences of adversity, the American Psychological Association [APA] (2014), suggested that resilience simply means “adapting well” to and “bouncing back” from adverse circumstances (p. 1). Researchers have identified many factors that potentially contribute to resilience, such as an individual’s personal characteristics, genetics/biology, positive family/social connections, and community factors (Gartland et al., 2019; Herrman et al., 2011). For example, Liu et al. (2020) found that youth who experienced high levels of adversity had better health outcomes when they also experienced high levels of protective factors in their lives, as compared to those who experienced adversity but did not have the benefit of mitigating protective factors. While these phenomena are well studied in the general population, there is little research specifically exploring the relationship between childhood adversity, resilience, and mental health among autistic individuals (Lai & Szatmari, 2019; Taylor & Gotham, 2016). Epidemiological research has shown that children on the autism spectrum endure more adversity than their non-autistic peers (Berg et al., 2016; Rigles, 2017) and experience a high prevalence of mental health diagnoses across the lifespan (Centers for Disease Control and Prevention [CDC], 2020; Joshi et al., 2010; Lai et al., 2019; Madden et al., 2017; Soke et al., 2018). While some researchers have explored the relationship between adversity and mental health challenges in children on the autism spectrum (e.g., Storch et al., 2013; Taylor & Gotham, 2016; Wood & Gadow, 2010), the current literature does not reflect the lived experiences of adversity and mental health among autistic individuals. Furthermore, limited research on the prevalence of protective factors in the lives of autistic children hasdescribed inconsistent findings. For example, Rigles (2017) found that children on the autism spectrum did not demonstrate as much resilience as their non-autistic peers, while McCrimmon et al. (2016) found no difference in the presentation of resilience factors among children on the autism spectrum and those without an autism diagnosis. It is important to note that the samples in these studies differed. McCrimmon et al. included only participants with a diagnosis of high-functioning autism, while Rigles included those who had received any autism-related diagnostic label. Therefore, these findings are not fully comparable. Regardless, this brings into question the role of resilience in mitigating the effects of adversity on autistic children. Rigles (2017) posited that the factors contributing to resilience in children on the autism spectrum may be different from what is currently being measured by researchers. Thus, more research is needed to understand the nature of resilience in autistic children.

### Aim

The lack of understanding of these phenomena among autistic individuals is concerning, particularly given the prevalence of poor mental health among autistic individuals. A conceptualization of the interplay between childhood adversity, resilience, and mental health in individuals on the autism spectrum would inform mental health promotion and intervention for this population, potentially improving their mental health. The aim of this study was to provide insight into the influence of childhood adversity on the lives of autistic adults, and the meaning of resilience in their lives.

### Theoretical and methodological approach

Autism is typically understood as a neurobiological condition characterized by (a) difficulty with social communication and (b) restricted, repetitive patterns of behaviour (National Institute of Mental Health [NIMH], 2020). Social communication challenges may lead to difficulty with relationships and managing changing social contexts (Lord & Jones, 2012; NIMH, 2020). Restricted, repetitive behaviours take many forms, sometimes presenting as physical movements, speech patterns, or a hyper-focus to certain activities or interests (NIMH, 2020). We sought to understand how being autistic interacted with childhood experiences of adversity and the development of resilience. We conceptualized autism as not only a diagnosis but also as part of participants’ identities, a notion rooted in neurodiversity (O’Dell et al., 2016). Proponents of n*eurodiversity* conceptualize autism as a neurobiological difference that contributes to unique strengths and abilities, rather than as a pathology to be treated or fixed (Baron‐Cohen, 2017; Silberman, 2015). Accordingly, neurodiversity and autistic identity were considered in interpreting the participants’ experiences of childhood adversity and resilience. We conceptualized mental health as being influenced by an interaction between autistic identity, adversity, and resilience.

Furthermore, autism-related research has most often been conducted by non-autistic researchers, leading to misunderstandings and misinterpretations of what it means to be autistic (Milton, 2014; Milton & Bracher, 2013; Milton et al., 2012). To decrease the risk of harm and make autism-related research more ethical, it is imperative that members of the autistic community, and other community stakeholders, be included in identifying priorities and informing methodologies (Chown et al., 2017; Milton & Bracher, 2013; Pellicano et al., 2014; Pellicano & Stears, 2011). This study incorporated philosophies and methods borrowed from participatory research to establish an inclusive research design and uphold the principles of ethical autism-related research (Chown et al., 2017; Pellicano, 2014).

Interpretative phenomenological analysis (IPA) informed this study, with participatory methods integrated throughout the design. IPA is rooted in phenomenology, which focuses on describing human experiences; hermeneutics, which seeks to interpret and make meaning of participants’ experiences; and idiography, by which researchers attend to the details and context of a participant’s experience (Smith et al., 2009). IPA researchers pay close attention to participants’ descriptions and understandings of their lived experiences, interpreting the participants’ interpretations of their own experiences. This makes IPA an appropriate methodology for conducting ethical autism-related research; through reflective interpretation, researchers do not claim to have first-hand knowledge of autistic experiences, rather they seek to understand and highlight the perspectives of participants, while acknowledging the influence of their own biases on the findings (MacLeod, 2019). This approach, along with positioning participants as experts, helps lessen the inherent power imbalances between non-autistic researchers and autistic participants (Howard et al., 2019). Our first step to increasing the participation of the autism community in this study was to engage advisors from the autism community (see Heselton et al., 2021).

## Methods

### Participants

To engage the community, we recruited five community members, three autistic individuals and two non-autistic mental health practitioners working within the autism community to provide input into the research design. Two advisors also identified as parents of children on the autism spectrum. Community advisors were interviewed via phone or videoconference. Community advisors provided recommendations for recruitment and data collection methods; and insight into autistic ways of thinking, communicating, and interacting, which influenced data generation and analysis. Following community engagement, participants were recruited through social media, local autism-community serving agencies, and a university online announcement board. Four young adults (aged 19–27), three women and one non-binary volunteered for this study. While the eligibility criteria for this study did not exclude participants based on gender, self-selection resulted in a participant sample that did not include any men. This allowed us to explore the unique experiences of women and non-binary participants, who are underrepresented in autism-related research (Lai et al., 2015).

### Procedure

The study design was approved by the institutional Research Ethics Board (Athabasca University, #23780). Both community advisors and participants reviewed the informed consent form and gave written or audio-recorded verbal informed consent prior to their interviews.

As per the advice of community advisors, participants were offered a variety of participation options (e.g., videoconference interview, phone interview, online chat interview, or written narratives with or without visual images). Two participants participated by phone, one opted for videoconference, and one engaged via online chat. In-person interviews were not offered due to COVID-19 safety concerns. The first author conducted the interviews. All participants opted to preview the interview guide. To enhance transparency and understanding, the interview guide provided detailed explanations of the purpose for each question and contained both open-ended questions and concrete, specific prompts (see supplemental data). Interview topics included childhood adversity, resilience, and mental health. Video and phone interviews ranged from 58 minutes to 107 minutes. Phone and video interviews were audio recorded and the online chat log was saved. Participants received a $25 gift card following the interview.

### Data analysis

As per IPA, each participant’s data were thematically analysed as a single case, and then analysis was completed across cases to identify common themes (Smith et al., 2009). In the initial noting phase, data were annotated according to descriptive, linguistic, and conceptual elements (Smith et al., 2009). Following initial noting of a single case, annotations and reflexive comments were reviewed to identify emergent themes. Similar themes were then clustered according to adversity or resilience. These themes were tabled, described, and then through abstraction, narrowed further to superordinate themes. Finally, the superordinate themes for each single case were compared, grouped, and renamed through abstraction, and then clustered one more time, described, and categorized into new superordinate themes. These final themes were tabled with supporting participant quotations to ensure themes derived across cases were found in the details of each participant’s recount of their experiences.

#### Rigour

To maintain rigour, the first author kept reflexive notes and integrated them into analysis. Reflexivity gives the IPA researcher opportunity to evaluate the influence of their own experiences on the analysis (Smith et al., 2009). To further demonstrate analytic rigour, the second author also completed initial noting and emergent theme development for each case, with ongoing discussions and critical evaluation throughout the process of thematization. Finally, the first author engaged participants a second time for the purpose of credibility checking. Participants were provided with a summary of overall themes and supporting quotations pertaining to their interview. Three participants provided feedback via recorded phone or videoconference, and one responded in writing. Through this process, participants confirmed aspects of the analysis that were consistent with their experiences. Often the themes resonated strongly with participants, and this compelled them to provide additional detail to enrich the description and understanding of their experiences. One participant also provided clarification when our interpretation of their experiences was not congruent with their interpretation. This additional data was incorporated into the findings. Additionally, participants chose a pseudonym to be identified by in the final report and dissemination. Participants were given another $25 gift card following the credibility check. The feedback was integrated into the findings.

## Findings

Themes of both adversity and resilience were apparent in the interpretations of participants’ recounted experiences. Themes related to adversity highlight the negative influence of adverse experiences on participants’ well-being, while resilience-related themes demonstrated protection from adverse experiences.

Through data analysis we derived 4 themes related to adversity and 3 themes related to resilience. Each theme encompassed multiple sub-themes, with a total of 27 sub-themes. In this section we will describe the over-arching themes related to adversity and resilience with quotes from participants related to important sub-themes.

### Adversity

Participants described diverse experiences of adversity including bullying at school by peers and teachers; rejection by peers; verbal, emotional, and physical abuse by parents; growing up in an oppressive communist dictatorship; and internal dysregulation and behaviour challenges. Despite their unique and varied experiences, participants described similar effects of adversity on their lives: *adversity influences social disconnection, adversity influences mental and emotional well-being, adversity influences sense of self*, and *adversity influences development into young adulthood.*

#### Adversity influences social disconnection

Participants identified social disconnection related to adversity, which affected their relationships with friends and romantic partners, even into young adulthood. While participants did not attribute their experiences of social disconnection to autistic characteristics, they were often victimized for being different, which exacerbated social disconnection. Social disconnection occurred in multiple ways, including through *avoidance of social interactions, and ostracization and stigmatization* by others.

For participants in this study, social disconnection occurred when participants chose to avoid social interactions based on previous negative experiences, such as bullying at school. Hannah said, “I just tried to stay away from [the other kids].” The social disconnection was not isolated to childhood: Chris described their ongoing avoidance of social connection, “I had a really hard time trusting people around me … and so, it’s become really hard for me to reach out to people on my own.”

Besides their own avoidance of social connection, participants described adverse experiences of ostracization, (i.e., exclusion from social groups and activities); and stigmatization, wherein they were negatively labelled and consequently mistreated by others, both of which led to social disconnection. Not surprisingly, participants were ostracized by peers. Hannah recalled being unwelcome among peers: “People were just telling me that I don’t deserve to be here.” Additionally, some participants were ostracized by adults. Shirley recounted an experience with her grandmother: “I started drumming on the table because I was so happy. [My grandmother] asked my mom to escort me out because I was disrupting her pets.”

Participants recounted a range of experiences of stigmatization. They described being negatively characterized and subsequently deemed unworthy and unwelcome. For instance, Hannah recounted the consequences of being labelled as unstable: “[I was] constantly being called a psycho, being beaten up at school.” The stigmatization Chris faced at school by peers was reinforced by teachers: “they were making a point of making sure that everybody knew that I was different and wrong. I had been built up as like this problem child who did everything wrong on purpose.” In a follow-up discussion, Saoirse expressed how policies and practices within the school system set her up to be ostracized and stigmatized:

Because I was in that spec ed/gifted class … we were still segregated from the rest, were still the different or the special kids. And while I’m really grateful that we had a specific place for us, why are we the ones that have to be treated as the other?

#### Adversity influences mental and emotional well-being

Participants in this study expressed a history of mental health challenges and emotional distress. These issues began in childhood and, for all four participants, continued into adulthood, with varying degrees of severity. Their experiences of childhood adversity particularly influenced *suicidal ideation* and *emotional distress*.

Suicidal ideation emerged as a common experience among participants. Chris’s suicidal thoughts and behaviours began at a young age: “I had first experienced suicidal ideation when I was eight … my first suicide attempt was when I was nine. It was rough.” Related to suicidal ideation was emotional distress. This included emotional pain, sadness, anxiety, anger, and self-hatred. Hannah experienced distress from a young age:

I hated myself since I was 5, actually …. I was really sad all the time. I was angry. I couldn’t control my anger …. When I was in high school [I] started therapy because my anxiety was really bad.

Shirley recalled, “As a child I was depressed a lot because I was always bullied at school or by my dad …. I’d say my mood was hurt and confused through my childhood.”

#### Adversity influences development into young adulthood

Saoirse frequently characterized her experiences of adversity as “formative,” and this attribution was similarly reflected in the other participants’ accounts. Formative experiences were those that influenced participants’ development, including how they interacted, behaved, and felt in adulthood. As they entered young adulthood, they had to *unlearn learned behaviours* and had *difficulty with adult relationships*.

The adversities that participants encountered were complex, ongoing, and pervasive. These experiences were interwoven with the development of their attitudes, beliefs, values, and behaviours. Over time, they learned that there were alternatives to these views and behaviours and, in some cases, that they were unhelpful or even unsafe. For example, Saoirse’s father modelled aggression:

I was using the same awful tactics that my father was. I was using aggression, and hatred and resentment, anger … I ended up just like my father …. And I was like, no, I can’t do that …. And that’s what made me look inside and do a lot more internal work.

Furthermore, participants experienced challenges with initiating and maintaining adult relationships. Shirley said, “I’ve also been in tons of failed relationships and recently got out of an abusive one which I feel is all linked to my past experiences.” Saoirse described how her template for interacting with others developed through interactions with her father:

Yeah, the relationships I had growing up in my family just did not give to me the tools … to communicate properly with people, to have a productive and positive conversation, to make friends, [or] to repair relationships.

#### Adversity influences sense of self

Participants held many negative perceptions of themselves and a lack of agency, which developed through feelings of *shame* and *powerlessness*.

Experiences of adversity taught participants that they should be ashamed of themselves because they were different and inadequate. Shirley tried desperately to hide her autistic identity from others:

Honestly, I felt very claustrophobic trying to hide who I was. Almost like you were trying to physically stuff me into a literal desk drawer at times …. I wanted to be normal and ‘non autistic’ growing up so I studied my peers to try and be like them.

Chris felt unworthy and inferior, “it was a lot of me internalizing that I was a bad person.”

Along with shame came feelings of powerlessness. Participants described feeling that they had no capacity to change their circumstances. Saoirse said of her conflict with her father, “it didn’t matter what I tried to do with my life …. it seemed insurmountable and I wasn’t going to be able to do anything, because I wasn’t allowed.” Hannah felt powerless to her emotional dysregulation, “I never meant to hurt anyone. It just happened because I lost control.”

### Resilience

Participants described factors in their childhoods that protected them from the effects of adverse experiences; resilience was interpreted as a function of these factors and evidenced in positive outcomes. In speaking about the meaning of resilience in their lives, participants identified internal and external factors that provided them with security, stability, and support in the face of adversity. Each participant defined resilience differently; however, their definitions alluded to a perseverance in the face of adversity and resistance to its potentially detrimental effects. From Saoirse:

Resilience, to me, means fighting back in the face of adversity … it sucked, but you had to deal with it, and you had to find a way to either overcome it, circumvent it, or cope with it.

Resilience factors were interpreted as being present in *places of refuge* and *identity*. Additionally, resilience influenced *transitions into young adulthood.*

#### Places of refuge

Resilience was fostered in safe spaces, where participants could escape from the ongoing and relentless stress of adversity in their lives—places where they were free to be themselves. These places of refuge were not only physical spaces, but also connection with other people and internal resources. Participants found refuges in external spaces—communities that were accepting and that gave participants a sense of belonging. Internal places of refuge included immersion in their *interests and talents* and their *imaginations*.

Participants found refuge and safety when they were accepted by others unconditionally and when they were able to feel a sense of belonging with others. Shirley recalled feeling safe with her aunt and cousin:

[My aunt] always made me laugh by being silly and so did my cousin who lived with her …. I remember in the summers we’d stay there he’d look through teen magazines with me, do the high school musical sing-alongs with me, etc., and my aunt was always pretending to be clumsier or sillier than she really was.

Saoirse highlighted her high school experience in a gifted programme:

Those kids were like me. That was really awesome. That was the first time that I had seen a conglomerate of classmates that had similar characteristics. And then I kind of realized, wow, I don’t have to cover up anymore, necessarily. I don’t have to wear a mask. I don’t have to try, and you know, spend the abhorrent amount of energy that I do trying to fit in in a neurotypical world. I could just be with these kids and it would be great.

Participants found internal refuge in the activities they were passionate about. Hannah expressed how she depended on her special interests for stability and security, “having a special interest to rely on … I really enjoyed lining up Little Pet Shops and organizing them. I acted out scenes from my life.” Saoirse described how her musical talent helped her, “if I needed to feel good … I would just sing and play the piano for hours and pretend. It was a really nice escape.”

Similarly, Chris recalled retreating into their imagination, as that was somewhere their hardships did not exist, “My favorite thing to do was to go to the pool and just swim. And I would like to imagine I was a fish or a mermaid or whatever because it was like an escapism thing.”

#### Resilience develops through identity

While adversity influenced participants’ sense of self and self-perception, their identities, namely their personal traits and uniqueness, also contributed to their resilience. The aspects of their identities that promoted resilience were *developing self-understanding, personal attributes, including determination*, and *a sense of pride*.

Related to adversity, participants talked about lacking self-understanding and being mystified by their own thoughts, feelings, and behaviours. Conversely, as participants developed self-understanding, they could integrate their uniqueness into a sense of identity, which influenced resilience. For example, Saoirse remembered identifying with Disney characters as a child:

Most people just think that I am a little bit more to myself, or … ‘strange’ or ‘eccentric’ or ‘weird’ are the terms that were used with me as a child … but I didn’t find anything wrong with being ‘eccentric’ or ‘strange’ …. I mean, frig, they called Belle in Beauty and the Beast strange and I was like, she’s not strange! She just likes to read books. I like to read books, too. What’s weird about that?

Hannah discussed how receiving an autism diagnosis changed her understanding of herself:

I got diagnosed when I was 17 years old and it was a really eye-opening experience because it finally explained what was going on in my childhood … I thought I had a personality disorder because I was so emotionally unstable, but it turned out to be just autism and that my sensory needs weren’t being met.

Personal attributes, such as Shirley’s sense of humour, contributed to participants’ overall sense of identity and resilience to adversity. Shirley said, “I’ve adapted a very dark sense of humor to my life experiences.” Additionally, several participants expressed their determination to make changes in their lives and circumstances and to be heard, despite the negative effects of adversity. As Saoirse’s understanding of her circumstances shifted, she gained the confidence to improve her situation:

Realizing that the problem wasn’t necessarily me … after that, I applied to university and college without [my father’s] permission. Because I was like, you know what? I will be damned if I am not allowed to go out and live my life.

Finally, developing a sense of pride in one’s accomplishments served as a resilience factor for participants. Hannah spoke about her school achievements, “I’ve always been a high achiever academically. I’m a straight A student and always have been. And I work really hard and really enjoy studying and that’s something that I’ve always taken pride in.”

#### Resilience influences transitions to young adulthood

For the participants in this study, resilience meant positivity and growth in their young adult lives, despite their experiences of adversity, which was evident as they developed *social connections* and demonstrated *moving forward* with their lives.

In young adulthood, participants expressed newfound social connections, including friendships and romantic relationships. Saoirse expressed gratitude for her current social connections, “I have amazing other supportive friends [and] my partner who I disclosed most of this information to. So, yeah, I’ve had really great people around.”

Participants were future-focused—making plans and engaging in activities, facing challenges with adaptability. Hannah happily noted how she had learned to regulate her emotions and behaviour, “On the whole, I am pretty much in control most of the time, I can control the sensory input. Saoirse described her life after leaving home, “I was able to explore the world … I started hanging out with people that I otherwise wasn’t allowed to hang out with. I started trying different activities.” Chris enrolled in a post-secondary programme and engaged in entrepreneurship, “[I’m] starting a small business with my friend.”

## Discussion

In this study, participants’ experiences of childhood adversity varied, however the effects were commonly destructive to their well-being and sense of self. Furthermore, there was commonality in their experiences of resilience. The influence of adversity in the lives of participants was not solely related to mental health, as has been identified in the research literature (Bellis et al., 2018; Bright et al., 2016; Rigles, 2017). Participants linked adversity to negative outcomes in other aspects of their lives, such as relationships and their social-emotional development into young adulthood. Related to resilience development, participants identified factors that were unique to their individual characteristics and circumstances but that had commonalities. Seeking refuge in safe people and places, as well as a sense of identity, were important for participants’ ability to manage the relentless adversity they faced.

Research has indicated a relationship between mental health symptomology and adversity in non-autistic children (Bellis et al., 2018; Bright et al., 2016; Rigles, 2017) and there is limited evidence that children on the autism spectrum also experience mental health challenges related to adverse experiences (Storch et al., 2013; Taylor & Gotham, 2016; Wood & Gadow, 2010), however these studies do not provide evidence from the lived experiences of autistic individuals. In a study by Ahlström and Wentz (2014) adolescents diagnosed with both autism and attention deficit hyperactivity disorder (ADHD) described stressors in their everyday lives that were similar to those of the participants in this study, including victimization by peers and adults, anxiety, and rejection. Our study is unique in that we not only described the presence of adversity and resilience in participants’ lives but did so by examining first-hand accounts from autistic adults. Therefore, this study provides insight into the immediate and long-term complex influence of adversity on their lives. Participants connected their mental health challenges, including depression, anxiety, and suicidal ideation, to adversity, symptomology that has been identified in previous research (Storch et al., 2013; Taylor & Gotham, 2016). Additionally, they described how adversity influenced other aspects of their lives—their diminished social interactions, lack of connection, and negative self-concept—and how it permeated their development beyond adolescence into young adulthood.

### Theoretical frameworks of adversity and resilience

Some theorists posit that the neurobiological characteristics of autism influence how adverse experiences are perceived by autistic children, thus mitigating or exacerbating their effects (Im, 2016; Kerns et al., 2015); however, this was not identified by participants in the present study as a feature of their experiences of adversity. Instead, these findings show a complex and contextual interaction of autism with both adversity and resilience that is simultaneously detrimental and protective. For example, autistic characteristics contributed to both adversity and resilience. Autism interacted with adversity to influence social disconnection, however not because autism was adverse in and of itself or as a confounding influence in participants’ interpretations of their experiences; rather this social disconnection was often prompted by maltreatment related to participants’ autistic traits and differences. This distinction does not preclude the damaging effects of these experiences on many aspects of the participants’ lives. In relation to resilience, participants’ autistic characteristics carried significance for them as resilience factors that mitigated the effects of adversity. Participants found safety and comfort in special interests and talents, as well as with peers who understood and experienced the world in a similar way.

Additionally, the present study goes beyond epidemiology and provides an in-depth exploration of the meaning of resilience in the lives of participants. In typically developing children, resilience is theorized to be the result of the mitigation of the negative effects of adversity by internal and external protective factors. Individuals who experience better health outcomes despite significant childhood adversity are considered resilient and this characteristic is attributed to the protection provided by common factors in their young lives (Gartland et al., 2019; Herrman et al., 2011; Liu et al., 2020; Moore & Ramirez, 2016). However, little is known about the characterization of resilience to adversity in children on the autism spectrum, as the available research is predominantly epidemiological and quantitative (e.g., McCrimmon et al., 2016; Rigles, 2017). In non-autistic children, factors such as supportive adults, personality characteristics, and positive social relationships have been identified as potentially protective against the negative effects of adversity (Gartland et al., 2019; Herrman et al., 2011). Autistic participants in the current study identified similar protective influences in their childhoods. They described positive internal and external influences on their lives, including personal attributes, attentive adults, and accepting social groups, but also elements unique to being autistic, such as special interests and talents, a sense of belonging among peers with similar traits and experiences, and self-understanding related to their autistic identities. Notably, participants also described some positive aspects of young adulthood, which suggests that resilience has mediated, to some degree, the negative effects associated with childhood adversity.

### Strengths, limitations, and implications for future research

The present study provides new insight into the experiences of childhood adversity and the meaning of resilience among autistic adults. While these findings need to be considered in the context of a small sample size, the sample is reasonable as per IPA methodology. Our findings offer nuanced attention to the details of participant accounts, thus providing an in-depth understanding of these phenomena in the lives of participants. Additionally, IPA provided an opportunity to hear directly from autistic individuals about their experiences, rather than drawing conclusions from non-autistic, outsider observations. This, along with integration of participatory methods into the study design (Heselton et al., 2021), aligns this study closely to the principles of ethical autism-related research (Chown et al., 2017).

Consistent with IPA, we engaged a small, homogenous sample of participants, which presented several limitations. Given that there were no male-identifying participants, the findings are somewhat constrained by gender; the experiences of men and boys may be different. However, given the gender bias towards men and boys in the diagnosis of autism, women and non-binary individuals are underrepresented in research (Lai et al., 2015), therefore this study addressed that gap.

Additionally, several community advisors suggested that limiting recruitment to individuals who had been diagnosed prior in childhood would exclude valuable input from those who went through childhood without a diagnosis. Future research comparing the experiences of autistic participants diagnosed in childhood rather than adulthood, or those self-diagnosed in adulthood, could provide understanding of the influence formal diagnosis has on experiences of childhood adversity and resilience.

Lastly, all participants had strong verbal communication skills and their accounts were not limited by expressive language differences. Given that differences in social communication and language use are commonplace for autistic individuals, the number of autistic individuals who are non or minimally speaking, and the high percentage of individuals on the autism spectrum with co-occurring intellectual disability, the results of this study may not be wholly representative of their experiences. This sub-set of the autistic population is understudied, and future research could explore their unique experiences of childhood adversity and resilience.

### Clinical implications

From the present study, we learned that childhood adversity has significant and long-term effects on the lives of autistic individuals, including their social connectedness, their emotional and mental well-being, their sense of self, and their development into adulthood. Furthermore, from these findings, it is evident that resilience plays an important role in providing safety and escape from adversity, maximizes individuals’ strengths to endure and overcome the adversity, and influences positive outcomes in early adulthood.

These findings compel clinicians and caregivers to pay attention to the potential sources of adversity for children, and to attune to the potentially ambiguous effects of those experiences and seek to foster resilience. This is similar to the conclusions of Ahlström and Wentz (2014), who suggested that clinicians give special consideration to the increased stress experienced by adolescents on the autism spectrum in coping with everyday life. Being alert and open to the potential root causes of behaviours, challenges with social interactions, and mental and emotional dysregulation could lead to earlier mitigative interventions fostering well-being, and even prevention of the effects of adversity. Furthermore, recognizing the unique attributes and activities of children on the autism spectrum that may be protecting them from the negative effects of adversity primes clinicians and caregivers to nurture the development of such traits and provide opportunities to engage in protective activities. Additionally, constructing external protective factors, such as mental health interventions, safe environments, and places of belonging and acceptance, may promote resilience in children on the autism spectrum.

When identifying and executing formal resilience-building interventions, it is important to recognize the potential pervasive and complex influence of adversity in the lives of autistic children and be aware that adversity affects more aspects of a child’s life than their emotional and mental well-being. There is ample evidence supporting resilience-building programmes in non-autistic children to improve their mental health (Dray et al., 2017). Additionally, some researchers have adapted resilience-fostering interventions for use with children on the autism spectrum, successfully fostering internal protective factors in these children (Mackay et al., 2017). However, for children on the autism spectrum, it is important to also address the social disconnection and harm to identity they may have suffered. Accordingly, providing external supports that minimize social disconnection and bolster a child’s sense of identity will be as important as promoting the development of internal resilience factors to improve mental health outcomes.

## Conclusion

These findings describe the pervasive and critical influence of childhood adversity on the lives of children on the autism spectrum and the unique, and often misunderstood, aspects of being autistic that may contribute to resilience. Their insights give a deeper understanding of how adverse childhood experiences affect young people and how better to support the development of resilience to minimize poor outcomes in adulthood. Clinicians and caregivers must be aware of the complex and pervasive effects of adversity on children on the autism spectrum, strive to minimize potentially adverse experiences, and work to foster internal and external resilience factors in these children.

## Supplementary Material

Supplemental Material

## References

[cit0001] Ahlström, B. H., & Wentz, E. (2014). Difficulties in everyday life: Young persons with attention-deficit/hyperactivity disorder and autism spectrum disorders perspectives. A chat-log analysis. *International Journal of Qualitative Studies on Health and Well-Being*, 9(1), 1–10. 10.3402/qhw.v9.23376 PMC4038720 24875238

[cit0002] American Psychological Association. (2014). The road to resilience. http://www.apa.org

[cit0003] Baron‐Cohen, S. (2017). Editorial perspective: Neurodiversity–a revolutionary concept for autism and psychiatry. *Journal of Child Psychology and Psychiatry*, 58(6), 744–747. 10.1111/jcpp.12703 28524462

[cit0004] Bellis, M. A., Hughes, K., Ford, K., Hardcastle, K. A., Sharp, C. A., Wood, S., Homolova, L., & Davies, A. (2018). Adverse childhood experiences and sources of childhood resilience: A retrospective study of their comorbid relationships with child health and educational attendance. *BMC Public Health*, 18(1), 1–12. 10.1186/s12889-018-5699-8 PMC6020215 29940920

[cit0005] Berg, K. L., Shiu, C., Acharya, K., Stolbach, B. C., & Msall, M. E. (2016). Disparities in adversity among children with autism spectrum, disorder: A population-based study. *Developmental Medicine & Child Neurology*, 58(11), 1124–1131. 10.1111/dmcn.13161 27251442

[cit0006] Bright, M. A., Knapp, C., Hinojosa, M. S., Alford, S., & Bonner, B. (2016). The comorbidity of physical, mental, and developmental conditions associated with childhood adversity: A population based study. *Maternal and Child Mental Health Journal*, 20(4), 843–853. 10.1007/s10995-015-1915-7 26694043

[cit0007] Centers for Disease Control and Prevention. (2020, September 25). Autism spectrum disorder (ASD): Data and statistics. http://www.cdc.gov

[cit0008] Chown, N., Robinson, J., Beardon, L., Downing, J., Hughes, L., Leatherland, J., Fox, K., Hickman, L., & MacGregor, D. (2017). Improving research about us, with us: A draft framework for inclusive autism research. *Disability & Society*, 32(5), 720–734. 10.1080/09687599.2017.1320273

[cit0009] Dray, J., Bowman, J., Campbell, E., Freund, M., Wolfenden, L., Hodder, R. K., McElwaine, K., Tremain, D., Bartlem, K., Bailey, J., Small, T., Palazzi, K., Oldmeadow, C., & Wiggers, J. (2017). Systematic review of universal resilience-focused interventions targeting child and adolescent mental health in the school setting. *Journal of the American Academy of Child & Adolescent Psychiatry*, 56(10), 813–824. 10.1016/j.jaac.2017.07.780 28942803

[cit0010] Felitti, V. J., Anda, R. F., Nordenberg, D., Willamson, D. F., Spitz, A. M., Edwards, V., Koss, M. P., & Marks, J. S. (1998). Relationship of childhood abuse and household dysfunction to many of the leading causes of death in adults: The Adverse Childhood Experiences (ACE) study. *American Journal of Preventative Medicine*, 14(4), 245–258. 10.1016/s0749-3797(98)00017-8 9635069

[cit0011] Gartland, D., Riggs, E., Muyeen, S., Giallo, R., Afifi, T. O., MacMillan, H., Herrman, H., Bulford, E., & Brown, S. J. (2019). What factors are associated with resilient outcomes in children exposed to social adversity? A systematic review. *BMJ Open*, 9(4), e024870. 10.1136/bmjopen-2018-024870 PMC6500354 30975671

[cit0012] Herrman, H., Stewart, D. E., Diaz-Granados, N., Berger, E. L., Jackson, B., & Yuen, T. (2011). What is resilience? *The Canadian Journal of Psychiatry*, 56(5), 258–265. 10.1177/070674371105600504 21586191

[cit0013] Heselton, G. A., Rempel, G. R., & Nicholas, D. B. (2021). Integrating community participation with interpretative phenomenological analysis: Reflections on engaging the autism community. International Journal of Qualitative Methods, 20, 1–10. 10.1177/16094069211055575

[cit0014] Howard, K., Katsos, N., & Gibson, J. (2019). Using interpretative phenomenological analysis in autism research. *Autism*, 23(7), 1871–1876. 10.1177/1362361318823902 30672307

[cit0015] Hughes, K., Bellis, M., Hardcastle, K. A., Sethi, D., Butchart, A., Mikton, C., Jones, L., & Dunne, M. P. (2017). The effects of multiple adverse childhood experiences on health: A systematic review and meta-analysis. *The Lancet Public Health*, 2(8), 356–366. 10.1016/s2468-2667(17)30118-4 29253477

[cit0016] Im, D. S. (2016). Trauma as a contributor to violence in autism spectrum disorder. *Journal of the American Academy of Psychiatry and the Law Online*, 44(2), 184–192. http://jaapl.org/27236173

[cit0017] Joshi, G., Petty, C., Wozniak, J., Henin, A., Fried, R., Galdo, M., Kotarski, M., Walls, S., & Biederman, J. (2010). The heavy burden of psychiatric comorbidity in youth with autism spectrum disorders: A large comparative study of a psychiatrically referred population. *Journal of Autism and Developmental Disorders*, 40(11), 1361–1370. 10.1007/s10803-010-0996-9 20309621

[cit0018] Kerns, C. M., Newschaffer, C. J., & Berkowitz, S. J. (2015). Traumatic childhood events and autism spectrum disorder. *Journal of Autism and Developmental Disorders*, 45(11), 3475–3486. 10.1007/s10803-015-2392-y 25711547

[cit0019] Lai, M. C., Kassee, C., Besney, R., Bonato, S., Hull, L., Mandy, W., Szatmari, P., & Ameis, S. H. (2019). Prevalence of co-occurring mental health diagnoses in the autism population: A systematic review and meta-analysis. *Lancet Psychiatry*, 6(10), 819–829. 10.1016/s2215-0366(19)30289-5 31447415

[cit0020] Lai, M. C., Lombardo, M. V., Auyeung, B., Chakrabarti, B., & Baron-Cohen, S. (2015). Sex/gender differences and autism: Setting the scene for future research. *Journal of the American Academy of Child & Adolescent Psychiatry*, 54(1), 11–24. 10.1016/j.jaac.2014.10.003 25524786 PMC4284309

[cit0021] Lai, M. C., & Szatmari, P. (2019). Resilience in autism: Research and practice prospects. *Autism*, 23(3), 539–541. 10.1177/1362361319842964 30971108

[cit0022] Liu, S. R., Kia‐Keating, M., Nylund‐Gibson, K., & Barnett, M. L. (2020). Co‐occurring youth profiles of adverse childhood experiences and protective factors: Associations with health, resilience, and racial disparities. *American Journal of Community Psychology*, 65(1–2), 173–186. 10.1002/ajcp.12387 31489651

[cit0023] Lord, C., & Jones, R. M. (2012). Annual research review: Re-thinking the classification of autism spectrum disorders. *Journal of Child Psychology and Psychiatry*, 53(5), 490–509. 10.1111/j.1469-7610.2012.02547.x 22486486 PMC3446247

[cit0024] Mackay, B. A., Shochet, I. M., & Orr, J. A. (2017). A pilot randomised controlled trial of a school-based resilience intervention to prevent depressive symptoms for young adolescents with autism spectrum disorder: A mixed methods analysis. *Journal of Autism and Developmental Disorders*, 47(11), 3458–3478. 10.1007/s10803-017-3263-5 28770525

[cit0025] MacLeod, A. (2019). Interpretative phenomenological analysis (IPA) as a tool for participatory research within critical autism studies: A systematic review. *Research in Autism Spectrum Disorders*, 64(2019), 49–62. 10.1016/j.rasd.2019.04.005

[cit0026] Madden, J. M., Lakoma, M. D., Lynch, F. L., Rusinak, D., Owen-Smith, A., Coleman, K. J., Quinn, V. P., Qian, Y. X., & Croen, L. A. (2017). Psychotropic medication use among insured children with autism spectrum disorder. *Journal of Autism and Developmental Disorders*, 47(1), 144–154. 10.1007/s10803-016-2946-7 27817163 PMC5328123

[cit0027] McCrimmon, A. W., Matchullis, R. L., & Altomare, A. A. (2016). Resilience and emotional intelligence in children with high-functioning autism spectrum disorder. *Developmental Neurorehabilitation*, 19(3), 154–161. 10.3109/17518423.2014.927017 24960312

[cit0028] Mehtar, M., & Mukaddes, N. M. (2011). Posttraumatic stress disorder in individuals with diagnosis of autistic spectrum disorders. *Research in Autism Spectrum Disorders*, 5(1), 539–546. 10.1016/j.rasd.2010.06.020

[cit0029] Milton, D. E. (2014). Autistic expertise: A critical reflection on the production of knowledge in autism studies. *Autism*, 18(7), 794–802. 10.1177/1362361314525281 24637428

[cit0030] Milton, D. E., & Bracher, M. (2013). Autistics speak but are they heard? *Medical Sociology Online*, 7(2), 61–69. http://www.medicalsociologyonline.org/

[cit0031] Milton, D., Mills, R., & Pellicano, L. (2012). Ethics and autism: Where is the autistic voice? Commentary on Post et al. *Journal of Autism and Developmental Disorders*, 44(10), 2650–2651. 10.1007/s10803-012-1739-x 23229456

[cit0032] Moore, K. A., & Ramirez, A. N. (2016). Adverse childhood experience and adolescent well-being: Do protective factors matter? *Child Indicators Research*, 9(2), 299–316. 10.1007/s12187-015-9324-4

[cit0033] National Institute of Mental Health. (2020, March 13). Autism spectrum disorder: Fact sheet. https://www.ninds.nih.gov

[cit0034] O’Dell, L., Bertilsdotter Rosqvist, H., Ortega, F., Brownlow, C., & Orsini, M. (2016). Critical autism studies: Exploring epistemic dialogues and intersections, challenging dominant understandings of autism. *Disability & Society*, 31(2), 166–179. 10.1080/09687599.2016.1164026

[cit0035] Pellicano, L. (2014). A future made together: New directions in the ethics of autism research. *Journal of Research in Special Educational Needs*, 14(3), 200–204. 10.1111/1471-3802.12070_5

[cit0036] Pellicano, E., Dinsmore, A., & Charman, T. (2014). Views on researcher-community engagement in autism research in the UK: A mixed-methods study. *PLoS One*, 9(10), 1–11. 10.1371/journal.pone.0109946 PMC4193852 25303222

[cit0037] Pellicano, E., & Stears, M. (2011). Bridging autism, science and society: Moving toward an ethically informed approach to autism research. *Autism Research*, 4(4), 271–282. 10.1002/aur.201 21567986

[cit0038] Rigles, B. (2017). The relationship between adverse childhood events, resiliency and health among children with autism. *Journal of Autism and Developmental Disorders*, 47(1), 187–202. 10.1007/s10803-016-2905-3 27807754

[cit0039] Silberman, S. (2015). *NeuroTribes: The legacy of autism and the future of neurodiversity*. Avery.

[cit0040] Smith, J. A., Flowers, P., & Larkin, M. (2009). *Interpretative phenomenological analysis*. Sage.

[cit0041] Soke, G. N., Maemer, M. J., Christensen, D., Kurzius-Spencer, M., & Schieve, L. A. (2018). Prevalence of co-occurring medical and behavioral conditions/symptoms among 4- and 8-year-old children with autism spectrum disorder in selected areas of the United States in 2010. *Journal of Autism and Developmental Disorders*, 48(8), 2663–2676. 10.1007/s10803-018-3521-1 29524016 PMC6041136

[cit0042] Storch, E. A., Sulkowski, M. L., Nadeau, J., Lewin, A. B., Arnold, E. B., Mutch, P. J., Jones, A. M., & Murphy, T. K. (2013). The phenomenology and clinical correlates of suicidal thoughts and behaviors in youth with autism spectrum disorders. *Journal of Autism and Developmental Disorders*, 43(10), 2450–2459. 10.1007/s10803-013-1795-x 23446993 PMC3808993

[cit0043] Taylor, J. L., & Gotham, K. O. (2016). Cumulative life events, traumatic experiences, and psychiatric symptomatology in transition-aged youth with autism spectrum disorder. *Journal of Neurodevelopmental Disorders*, 8(1), 1–11. 10.1186/s11689-016-9160-y 27468315 PMC4962443

[cit0044] Wood, J. J., & Gadow, K. D. (2010). Exploring the nature and function of anxiety in youth with autism spectrum disorders. *Clinical Psychology: Science and Practice*, 17(4), 281–292. 10.1111/j.1468-2850.2010.01220.x

