# Supplementary material for: “Realizing the problem wasn’t necessarily me”: the meaning of childhood adversity and resilience in the lives of autistic adults
Source: Int J Qual Stud Health Well-being. 2022 Mar 17;17(1):2051237. doi: 10.1080/17482631.2022.2051237 (PMC8933012; doi:10.1080/17482631.2022.2051237)
Supplement: Supplemental Material [file ZQHW_A_2051237_SM4714.docx]

**PARTICIPANT INTERVIEW GUIDE**

**Understanding Childhood Adversity and Resilience: Perspectives of Autistic Adults**

There are 13 questions, with most having multiple sub-questions. Please read the following instructions before starting.

**Instructions**

1. Most of these questions are open-ended and invite you to reflect on specific aspects of your experiences. Many of the questions are followed by more specific sub-questions. If a sub-question does not feel true for your experience, please indicate that, and tell me why it does not apply.
2. Some questions have prompts in italics. It is not necessary that you answer these prompts directly, however, if you are struggling to respond to the question, you may refer to the prompts to focus your ideas. Use as many or as few of the prompts as you would like to aid you in responding to the question.
3. You may write as much as you want for each answer.
4. When you have completed the entire questionnaire, please return it to me by email.

**Questions**

The purpose of these questions is to get to know you and understand the context you grew up in.

**Tell me about your childhood.**

- 1. Where did you grow up?
  2. What was the make up of your family?
  3. What kind of elementary school/program did you attend? What kind of junior high did you attend? What kind of high school? (*Prompt: **you do not have to answer these questions unless you choose to. They are simply prompts to help you decide what to say in response to this question** Were you included in a regular classroom? Did you attend a special program or classroom for parts or all of your day?)*
  4. What kind of things did you like to do in your free time as a child?
  5. Tell me about any lessons or extracurricular activities that you participated in as a child.
  6. Tell me about your childhood pets.

1. The purpose of this question is to develop an understanding of how you characterize your current emotional and mental well-being, as later I will be asking how childhood experiences may have influenced your mental health.

**How would you describe your current mental health?**

*(Prompts: Do you have any co-occurring mental health diagnoses? If yes, what are they and how do you feel about those diagnoses? Do you have concerns about your mental health (whether you have a formal diagnosis or not)? What is your mood like? How do you feel when you wake up in the morning? How do you think your mental health could be better than it is? How does your mental health impact your day-to-day functioning? How do you manage your mental health? What do you think would help you? What supports do you wish you had?*

1. The purpose of this question is to understand your challenges with mental health as a child, and to identify any supports you may have had in place. Again, this relates to understanding the influence of your childhood experiences on your mental health at the time.

**How would you describe your mental health as a child?**

- 1. What was your mood like as a child?
  2. What was your mood like as an adolescent?
  3. Tell me about any diagnosed mental health issues in childhood or adolescence.
  4. How did you feel about those diagnoses at the time?
  5. How do you feel about those diagnoses now?
  6. Tell me how you managed your mental health at that time?
  7. Who supported you and how?
  8. What supports do you wish you had?

1. The purpose of this question is to make sure I understand the meaning you attribute to the word adversity. If you and I have different definitions of adversity, then we will not understand each other as we proceed with understanding your experiences together. Your answer may also provide insight to how adversity may be experienced by autistic children similarly to or differently from neurotypical children.

**What does the term adversity mean to you?** *(Prompts: tell me what the word adversity makes you think of? What is another word you would use to describe adversity? What would you imagine if somebody told you they had experienced adversity?)*

1. The purpose of these questions is for me to develop a picture in my mind of how you experienced adversity as a child, and what the wholistic impact of that adversity was on you. I also want to know how you view yourself in the context of that adversity.

**Tell me about a specific experience from your childhood that you would consider to be adverse.**

- - 1. How old were you?
    2. Where did this experience occur (e.g., school, home)?
    3. Who else was involved?
    4. Describe the incident.
    5. How often did this, or a similar incident, occur?
    6. How did you feel at the time?
    7. How did this adverse experience impact your behaviour?
    8. How did this adverse experience impact your relationships?
    9. How did this adverse experience impact your feelings about yourself?
    10. What did you do in the adverse situation?
    11. Tell me about anyone who may have helped you in that adverse situation. Who were they?
    12. Did you hide your feelings about this experience from others? If so, how did you do that? Why did you hide your feelings?

1. The purpose of these questions is to understand the long-term influence of childhood adversity in your life.

**Thinking about that adverse experience, how has it influenced your life now?**

- - 1. How do you feel about that experience now?
    2. How often do you think about that experience?
    3. How has that childhood experience affected your feelings now?
    4. How has that experience affected your behaviour now?
    5. How has that experience affected how you feel about yourself now?
    6. How has that experience affected your relationships now?
    7. How has that experience affected your daily life now?
    8. How has that experience affected your mental health now?
    9. How has that experience influenced how you interact with others now?
    10. How has that experience influenced your adult relationships?
    11. Has that experience had any other effects on you that you would like me to know about?

1. The purpose of this question, again, is to make sure that I understand what resilience means to you. This will give us a common language for our discussion of resilience. Your answer to this question might also provide insight into how your understanding of resilience may be similar to or different from the neurotypical perspective.

**What does resilience mean to you?** *(Prompts: what do you think of when you hear the word resilience? What is another word you would use to describe resilience? What would you imagine if somebody you were speaking with used the term resilience?*

1. The purpose of these questions is to help me understand what resilience factors you have within you that may have helped you get through an adverse experience. I also want to understand the meaning of those factors for your well-being and your life.

**What do you think it is about you that helped you get through that adverse situation you described earlier?** (*Prompts: what are some traits you possess that helped you in that situation? Give me an example of something you did in that situation that helped you?)*

- - 1. How did you feel about that aspect of yourself at the time?
    2. How do you feel about that aspect of yourself now? Why?
    3. Did you realize at the time that you were helping yourself? If you did, how did you know?
    4. What do you do as an adult to help you get through hard things?

1. The purpose of this question is to determine if you view the internal factors you described in the last question as something that built resilience in you.

How do you think that these traits helped or did not help to lessen the impact of the adverse experience you described?

- - 1. What were some positive results of u using those aspects of yourself in the adverse situation, if any?
    2. What were some negative effects of using those aspects of yourself in the adverse situation, if any?

1. The purpose of these questions is to understand what factors outside of yourself were influential in helping you get through adversity and what influence they may have had on your well-being and your life.

**Tell me about who or what in your life helped you get through the adverse experience you described.**

- - 1. How did you feel about that person or thing now?
    2. How does your experience with that person/thing in the past influence your life now?
    3. Do you think that this person or thing helped lessen the effects of the adverse experience you described? If so, how?

1. The purpose of this question is to understand what safety looked like for you as a child. I am curious if you felt safe as a child and if so, what contributed to that feeling. I am also curious if safety plays a role in the impacts of adversity and /or resilience.

**Describe a place that you could go to feel safe as a child.**

- - 1. Was the safe space internal or external (e.g., a physical place or somewhere you would go in your mind?)?
    2. Who else, if anyone, was in this space?
    3. How did you feel when you were in this space?
    4. Did you access this safe space when you experienced the adverse experience you described above?
    5. How did being in your safe space help you?

1. The purpose of this question is to understand how you viewed your autism diagnosis and how you view it now. I am curious how being autistic interacted with your experiences of adversity to influence your emotional and mental well-being.

**How did you view your autism diagnosis as a child?**

- - 1. What was the influence of autism on your experience of adversity?
    2. What was the influence of adversity on the role of autism in your life?
    3. Do you think having an autism diagnosis and/or autistic identity influenced how you felt about the adverse experience at the time?
    4. Do you think your autism diagnosis and/or autistic identity influenced how you viewed yourself at the time?
    5. How do you think your autism diagnosis and/or autistic identity influenced how you managed the adverse experience?
    6. How do you think your autism diagnosis and/or autistic identity influences your current mental health?

1. The purpose of this question is to create space for you to share anything else you want me to know.

**Is there anything else you would like me to know about your experiences?**

1. The purpose of this question is to give me feedback on the experience of participating in this study so that I can improve these types of experiences for participants in the future.

**How was this experience of participating in research for you?**

**Final Thoughts**

Thank you again for providing me with your thoughtful and honest answers to these hard questions. I am so grateful for your willingness to participate in my study and honouring me with your stories. There are a few final things I would like to remind you of:

- I will contact you in a few days to follow-up on our conversation, to give you a chance to ask any questions or tell me anything else you think is important. What is the best way to contact you?
- If answering these questions has brought up difficult emotions and thoughts, please reach out to your support systems and make sure you do the things you normally do to take care of yourself. Your well-being is the most important thing to me!
  - You have access to two free counselling sessions. You are welcome to see any counsellor you choose, or I can refer you to someone. You can pay for the session yourself and send me the receipt. I will e-transfer you the amount you paid. Alternatively, you can ask the therapist to contact me and I will pay them directly. They do not need to reveal your name to me. You have six months following our interview to claim this.
  - I will follow-up with you in 4-8 weeks so that you can offer feedback on the accuracy of my understanding of what you have shared with me.
